# A Role for Homeostatic Drive in the Perpetuation of Complex Chronic Illness: Gulf War Illness and Chronic Fatigue Syndrome

**DOI:** 10.1371/journal.pone.0084839

**Published:** 2014-01-08

**Authors:** Travis J. A. Craddock, Paul Fritsch, Mark A. Rice, Ryan M. del Rosario, Diane B. Miller, Mary Ann Fletcher, Nancy G. Klimas, Gordon Broderick

**Affiliations:** 1 Center for Psychological Studies, Nova Southeastern University, Fort Lauderdale, Florida, United States of America; 2 Graduate School for Computer and Information Sciences, Nova Southeastern University, Fort Lauderdale, Florida, United States of America; 3 Institute for Neuro-Immune Medicine, Nova Southeastern University, Fort Lauderdale, Florida, United States of America; 4 Department of Medicine, Faculty of Dentistry and Medicine, University of Alberta, Edmonton, Alberta, Canada; 5 Centers for Disease Control and Prevention, National Institute for Occupational Safety and Health, Morgantown, West Virginia, United States of America; 6 Department of Medicine, Miller School of Medicine, University of Miami, Miami, Florida, United States of America; 7 College of Osteopathic Medicine, Nova Southeastern University, Fort Lauderdale, Florida, United States of America; 8 College of Pharmacy, Nova Southeastern University, Fort Lauderdale, Florida, United States of America; University of Tennessee, United States of America

## Abstract

A key component in the body's stress response, the hypothalamic-pituitary-adrenal (HPA) axis orchestrates changes across a broad range of major biological systems. Its dysfunction has been associated with numerous chronic diseases including Gulf War Illness (GWI) and chronic fatigue syndrome (CFS). Though tightly coupled with other components of endocrine and immune function, few models of HPA function account for these interactions. Here we extend conventional models of HPA function by including feed-forward and feedback interaction with sex hormone regulation and immune response. We use this multi-axis model to explore the role of homeostatic regulation in perpetuating chronic conditions, specifically GWI and CFS. An important obstacle in building these models across regulatory systems remains the scarcity of detailed human *in vivo* kinetic data as its collection can present significant health risks to subjects. We circumvented this using a discrete logic representation based solely on literature of physiological and biochemical connectivity to provide a qualitative description of system behavior. This connectivity model linked molecular variables across the HPA axis, hypothalamic-pituitary-gonadal (HPG) axis in men and women, as well as a simple immune network. Inclusion of these interactions produced multiple alternate homeostatic states and sexually dimorphic responses. Experimental data for endocrine-immune markers measured in male GWI subjects showed the greatest alignment with predictions of a naturally occurring alternate steady state presenting with hypercortisolism, low testosterone and a shift towards a Th1 immune response. In female CFS subjects, expression of these markers aligned with an alternate homeostatic state displaying hypocortisolism, high estradiol, and a shift towards an anti-inflammatory Th2 activation. These results support a role for homeostatic drive in perpetuating dysfunctional cortisol levels through persistent interaction with the immune system and HPG axis. Though coarse, these models may nonetheless support the design of robust treatments that might exploit these regulatory regimes.

## Introduction

The hypothalamic-pituitary-adrenal (HPA) axis, a key component in the body's stress response, serves to articulate changes in a broad range of homeostatic regulators as a function of environmental cues. Such cues can consist of both physical stressors (injury, infection, thermal exposure) and psycho-emotional stressors (frustration, fear, fight or flight decisions). Instantiation of this survival program is accomplished through controlled modulation of the neuroendocrine and immune systems, as well as the sympathetic nervous systems [1]–[3]. Considering its function as a broad-reaching integrator of major physiological systems, it is no surprise that numerous chronic conditions have been associated with abnormal regulation of the HPA axis, including major depressive disorder (MDD) [4], [5], post-traumatic stress disorder (PTSD) [6]–[8], Alzheimer's disease [9], Gulf War Illness (GWI) [10]–[12], and chronic fatigue syndrome (CFS) [13]–[15]. When compared to non-deployed veterans, Golier et al. [10] found that symptomatic Gulf War veterans without psychiatric illness, as well as veterans with PTSD alone, showed significantly greater cortisol suppression to dexamethasone (DEX) suggesting markedly enhanced negative feedback along the HPA axis. Further study by these same investigators indicated that this might be due to a significantly attenuated ACTH response by the pituitary in veterans with GWI without PTSD [11], [12]. A similar suppression of cortisol response to DEX was found in CFS subjects by Van Den Eede et al. [13] with this being further exacerbated by oestrogen intake. With regard to HPA circadian dynamics, CFS subjects were found to exhibit significantly increased adrenal sensitivity to ACTH and marginally increased inhibitory feedback during the nocturnal period when compared with control subjects and CFS subjects comorbid with fibromyalgia (FM) [14], [15]. Conversely the pain-dominant CFS-FM subjects showed significantly blunted cortisol inhibitory feedback. While evidence such as this implicates abnormal regulation of HPA function leading to chronic hypocortisolic and hypercortisolic states in these illnesses, the genesis of this dysregulation is unclear.

**Figure 1:**
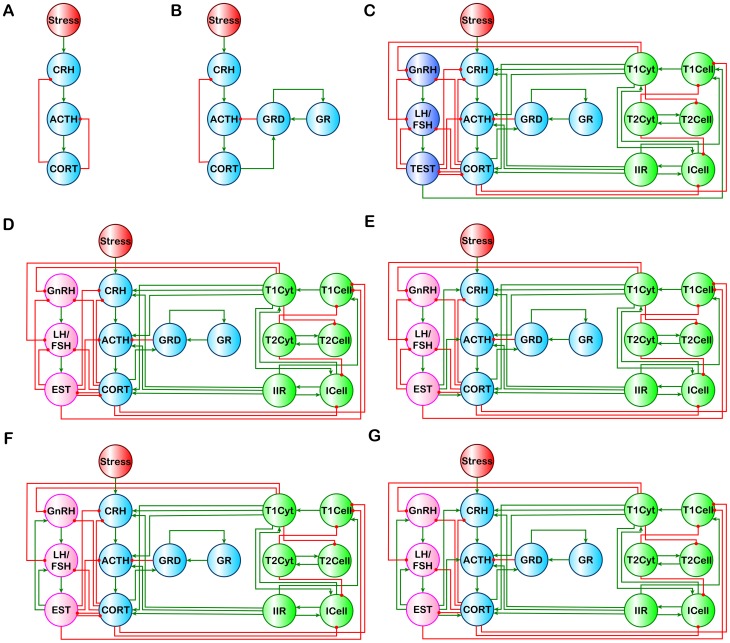
Standard and extended HPA models. (A) Standard HPA model. (B) HPA-GR model of Gupta et al. [22]. Integrated models (C) HPA-GR-Immune-HPGa for males, and (D) HPA-GR-Immune-HPGb, (E) HPA-GR-Immune-HPGc, (F) HPA-GR-Immune-HPGd, and (G) HPA-GR-Immune-HPGe for females. For (C) – (G) connections between the sex steroid EST and the HPG and HPA components change between stimulatory and inhibitory to capture the effects of the menstrual cycle.

Previously we investigated the possibility that some of these pathological states may coincide with naturally occurring alternate homeostatic stable states [16]. These “backup programs” would offer a way of maintaining homeostatic control in crisis situations at the cost of reduced function. The existence of such multiple stable states is characteristic of systems that incorporate feed-forward and feedback mechanisms. Feed-forward loops in biology play the crucial role of driving rapid acute responses, while feedback loops will generally limit the extent of a response. Both will also drive complex dynamic behavior, including differentiation and periodicity [17]. While small perturbations may force temporary departures, these systems return to their original resting states once these perturbations are removed. If however, the perturbation is of significant strength and duration, the system may be incapable of returning to its normal operating regime and instead may assume a new alternate resting state. Knowledge of the system dynamics can allow us to map these different stable states and several mathematical models of the HPA exist [16], [18]–[22]. So far, only one such model is known to accommodate multi-stability in the dynamic behavior of the HPA axis. It does so via the addition of a feed-forward mechanism involving dimerization of the glucocorticoid receptor (GR) complex [22] (Figure 1). In this process glucocorticoid (GC) bound GRs form homodimers that translocate into the cell nucleus to bind DNA, up-regulating GR synthesis and producing a positive feedback loop. However, this model and the majority of other models do not extend beyond the physiological boundaries of the HPA axis itself and thus are limited in their predictive capabilities. As discussed in the following sections, HPA activity is intertwined with the behavior of the hypothalamic-pituitary-gonadal (HPG) axis and the immune system, among others, and this interplay should not be ignored when considering the number and nature of stationary states available to the overarching system. Our hypothesis is that these alternate regulatory regimes may facilitate the persistence of complex chronic illnesses like GWI and CFS. To evaluate the role of alternate homeostatic attractors in these illnesses we constructed a computational model of regulatory control linking the HPA, HPG and immune systems.

There is a substantial body of physiological and biochemical data for many biological systems describing the connectivity between molecular and cellular elements, the presence of recurring structural motifs and functional modules. For example, negative autoregulation, in which a transcription factor represses its own transcription, is a simple network motif observed in many transcription networks. While, numerous motifs have been found in biological networks (negative/positive autoregulation, coherent/incoherent and multi-output feed-forward loops, single-input modules and dense overlapping regulons) [17], data regarding the precise stoichiometry and kinetics within and between these multiple systems in humans is extremely limited. Obtaining many of these parameters in humans currently presents significant challenges associated with invasive sampling. As such, many existing models rely heavily on animal data as a source of kinetic parameters, or adopt general order of magnitude estimates when this data is unavailable. Using such estimates allows Ordinary Differential Equation (ODE) based models to provide detailed descriptions of transitory behavior, albeit for well-characterized systems. To broaden this scope and draw on the rich body of known molecular and cellular interactions in physiological and biochemistry, we have adopted the discrete logical network methodology proposed originally by Thomas et al. [23], [24] and developed further by Mendoza and Xenarios [25]. By applying logic rules to a network of known interactions it is possible to identify the number of stable resting states, their type as well as their molecular and cellular description, without detailed knowledge of the response dynamics. Here detailed kinetic data is not required as working with connectivity alone allows useful qualitative insights regarding the stability of these systems. In this work we use this method to extend our previous analysis of human HPA axis dynamics by including its regulatory interactions with the neighboring HPG axis and immune system. This resulting mathematical model better represents the complexity of endocrine-immune interactions by supporting the detection and identification of alternate resting modes of the HPA-HPG-immune axis. Based on connectivity information alone, we show that multi-stability is easily obtained from these interacting systems. Moreover, we show that experimental data from our on-going studies of GWI and CFS show better alignment with these alternate resting modes than with the typical healthy homeostatic stable state. Ultimately, knowledge of such homeostatic modes could be used to identify promising applications of pharmaceutical, hormone and/or immune therapy that exploit the body's natural dynamics to reinforce treatment effects.

## Methods

### Ethics Statement

All subjects signed an informed consent approved by the Institutional Review Board of the University of Miami. Ethics review and approval for data analysis was also obtained by the IRB of the University of Alberta.

### An Integrative Multi-systems Model of the HPA-HPG-Immune System

There is a substantial amount of physiological data describing the HPA, HPG and immune systems as stand-alone entities. To a much lesser degree there also exists evidence for the mutual interactions between these systems. The following sections describe the experimental evidence used to infer the topology of an overarching HPA-HPG-immune interaction network (Figure 1).

#### The HPA Axis

Activation of the HPA axis begins at the paraventricular nucleus (PVN) of the hypothalamus. Specifically, afferents transmitting stress related information in the brain converge on the medial parvocellular neurons of the PVN inducing the release of several peptides, including corticotropin-releasing hormone (CRH) and arginine vasopressin (AVP), into the pituitary hypophysial-portal circulation. The unique vascular system allows very small quantities of these hypothalamic hormones to act directly on their targets in the anterior pituitary without dilution by systemic circulation. CRH and AVP act in conjunction on membrane bound CRH-R1 receptors in the anterior pituitary to stimulate adrenocorticotropic hormone (ACTH) synthesis, and its rapid release into peripheral circulation. ACTH circulates to the adrenal cortex where it acts on the membrane bound MC2-R receptor to simulate the release of GCs (corticosterone in the rat, and cortisol (CORT) in humans and nonhuman primates). To regulate the stress response, GCs exert negative feedback at the hypothalamus and pituitary to inhibit further synthesis and release of CRH and ACTH, respectively [26]. This is the standard view of the HPA axis utilized in the majority of models (Figure 1 A). However, as noted by Gupta et al. [22] circulating glucocorticoids act via cytostolic GRs, which, unlike membrane bound receptors, dimerize (GRD) and translocate into the cell nucleus upon activation to up-regulate GR synthesis and interact with other relevant transcription factors, or GC-sensitive genes (Figure 1 B). Gupta et al. included this GR expression feed-forward loop at the pituitary, as it is a main driver of the HPA axis, and found a resulting bistability in the HPA system [22]. However, all nucleated cells possess GRs, as GCs influence practically every system in the body, suggesting this feed-forward loop may be important in other tissues beyond the HPA axis. As described below major systems affected by GCs include the HPG axis and the immune system.

#### The HPG Axis

GCs have an inhibitory effect on the HPG axis, a central regulator of the reproductive system, at all levels [27]–[31]. Activation of the HPG starts from brain generated pulsatile signals that stimulate the preoptic area of the hypothalamus to produce gonadotropin-releasing hormone (GnRH). GnRH is secreted into the pituitary hypophysial portal bloodstream, which carries it to the pituitary gland, where it activates membrane bound GnRH-R receptors, resulting in the synthesis and secretion of luteinizing hormone (LH) and follicle-stimulating hormone (FSH) into circulation. These gonadotropins flow to the gonads where they work synergistically to promote the secretion of the sex steroids. In males, LH binds to receptors on Leydig cells in the testes to stimulate the synthesis and secretion of testosterone (TEST). In females, LH activates receptors on Theca interna cells in the ovaries to stimulate the release of androstenedione, which is aromatized by granulosa cells to produce estradiol (EST), and progesterone (PROG). TEST negatively feeds back on the HPG to inhibit GnRH, FSH and LH secretion and synthesis [27]. This feedback mechanism is somewhat more complex in females where, depending on the phase of the female menstrual cycle, EST and PROG can exert either positive or negative feedback on the production and release of GnRH and the gonadotropins [30], [32], [33].

A lesser-known aspect is that several components of the HPG axis exert reciprocal effects on the HPA axis [27], [28], [30]. Testosterone exhibits an inhibitory effect on all levels of the HPA [27] (Figure 1 C), whereas EST and PROG can serve to stimulate or inhibit the HPA axis depending on menstrual cycle phase, or phase of life [28]. These affects may be mediated through changes in adrenocorticoid synthesis, stress-induced ACTH and GC release, and CRH and AVP synthesis in the PVN, by direct activation of oestrogen and androgen receptors along the HPA or via interaction between GRs and sex steroid receptors to regulate transcription [27], [28], [30]. Thus, an interactive functional crosstalk exists between the HPA and HPG axes, which cannot be ignored when investigating HPA axis regulation and dysfunction. Mutual inhibition between the HPA and HPG (Figure 1 C) was considered standard for males. However, as it is not clear whether the EST and PROG inhibition/stimulation of the HPA occurs in coordination with the inhibition/stimulation of the HPG, these cases were explored for females alone as separate alternative models of the HPA-HPG interaction (Figure 1 D–G) in addition to the model considered for males.

#### A Simple Model of the Immune System

While not typically considered part of the neuroendocrine system, the immune system plays a very important role in regulating the HPA axis. Here we base our simplified immune system upon our previous work detailing the communication network of the immune response [34]. Cells of the innate immune response (ICells), including mononuclear phagocytes, such as macrophages, and dendritic cells, natural-killer (NK) cells, endothelial cells and mucosal epithelial cells, communicate via the release of numerous cytokines. Cytokines that regulate the innate immune response (IIR) include interleukin (IL) -1, IL-6, IL-8 and tumor necrosis factor alpha (TNF-α), and can also include IL-12, a primary mediator of early innate immunity. Primarily, these signals serve to activate and recruit other ICells, which in turn produce more cytokines. IL-15, which stimulates proliferation of NK cells and effector T-lymphocytes, can also be considered as part of the IIR as well as IL-23, an important inflammatory signal contributing to the Th17 response against infection.

IIR signals can also serve to prime helper T cells towards a Th1 type adaptive immune response (T1Cell). This response produces Th1 proinflammatory cytokines (T1Cyt) including IL-2, interferon-gamma (IFN-γ), and tumor necrosis factor beta (TNF-β), which further activate ICells, while suppressing the Th2 adaptive immune response (T2Cell). The T2Cell is responsible for the production of the Th2 anti-inflammatory cytokines (T2Cyt) IL-4, IL-5, IL-10 and IL-13, which have important anti-inflammatory and immunosuppressive activities, and serve to inhibit the activity of T1Cell and ICells.

Cytokines can also serve as mediators between the immune and endocrine systems. Between the HPA and the immune network there exists a mutual crosstalk [35]–[37] (Figure 1 C–G). The IIR and T1Cell cytokines selected here serve to stimulate the HPA axis at all levels [35]–[37]. CORT, in turn, acts to suppress the activity of ICells (specifically NK cells [38], and DC cells [39]), and the T1Cell [40] causing a shift from the inflammatory to the anti-inflammatory response [35], [36], [41]. The interaction between the HPG and the immune system is complex and sexually dimorphic, and is still an active field of research. However, at a general coarse level of description TEST serves to stimulate the development of the Th1 response [42] (Figure 1 C), whereas EST inhibits the Th1 response causing a shift towards the Th2 anti-inflammatory response [42], [43]. The reciprocal crosstalk from the immune system to the HPG is equally intricate. In broad terms this conversation is communicated via T1Cyt. Receptors for TNF-α and IFN-γ are expressed in testicular Leydig cells and there is evidence that these cytokines can directly inhibit testosterone production [44]. TNFα also decreases the release of GnRH in the hypothalamus and LH in the pituitary gland in both males [44] and females [45] eventually leading to a decrease in sex steroid levels. As such, we model the T1Cyt as inhibiting GnRH and LH/FSH in both male and female models.

### A Discrete State Representation

Following the methods of Thomas et al. [23], [24], and more recently Mendoza and Xenarios [25], the neuroendocrine-immune system was represented as a connectivity model consisting of interconnected molecular and cellular variables with three discrete states: −1 (inhibited), 0 (nominal) and 1 (activated). According to this type of model the current state of all variables in a system is described by a state vector 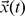, such that:
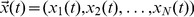
(1)where 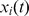 is the state of the *i^th^* variable of the *N* variable system at time *t*. The image vector 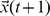 describes the preferred state towards which the system evolves in the next time increment. The state value of the image vector for the *i^th^* variable is determined from its current state and a set of balanced ternary logic statements based on the current value of variable and the mode of action (i.e. activate or inhibit) of the neighboring input variables. These logic statements are expressed as follows (Eq. 2):
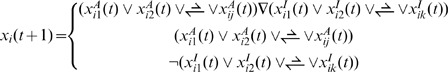
(2)where the ∇, ∨, and ¬ symbols are ternary HIGH/LOW PASS, OR and NOT operators, 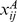 is the state of the *i^th^* variable's *j^th^* activator, 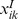
is the state of the *i^th^* variable's *k^th^* inhibitor. The ternary operators given in Equation (2) are described in further detail in Table 1, Table 2, Table 3. The first entry in Equation (2) is used when the variable possesses activators and inhibitors, the middle when the variable has only activators and last when the activator has only inhibitors.

**Table 1:** Ternary HIGH/LOW PASS operator.

A ∇B	B = −1	B = 0	B = 1
**A = −1**	0	0	−1
**A = 0**	0	0	−1
**A = 1**	1	1	0

**Table 2:** Ternary OR operator.

A∨B	B = −1	B = 0	B = 1
**A = −1**	−1	0	1
**A = 0**	0	0	1
**A = 1**	1	1	1

**Table 3:** Ternary NOT operator.

A	¬A
**−1**	1
**0**	0
**1**	−1

Applying Equation (2) to each variable in the model for the *m^th^* state of the system, 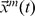, defines the image vector 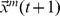 for that state. With 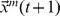 defined, the system may be updated asynchronously (allowing only one variable to change at a time) following the generalized logical analysis of Thomas et al. [23], [24]. According to this method the *i^th^* variable of the *m^th^* state vector 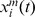 is moved one step towards its preferred image 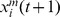 (e.g. If 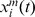  = −1 and 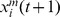  = 1, then 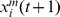 is set to 0). Thus, for each current state of the system there are potentially several subsequent states towards which it may asynchronously evolve.

The number of states, and the values they can be assigned, determine the total number of states available to the model system. With the ternary logic used here, a model of *N* variables possesses *3^N^* states. As a result, the number of states increases rapidly as new variables are added. By analyzing all possible states of the system a temporal sequence of states may be discerned. To interpret the results, each state of the system can be represented as an element in a graph. The evolution from one state to a subsequent state can be represented as a directed edge between the two states in this graph. Representation of the state trajectories in this fashion makes it possible to draw on the concepts and tools of graph theory for analysis of the system dynamics. Steady states are defined as those states for which the image vector is the same as the current state vector; in other words the state possesses an out degree of 0.

### Experimental Data

Experimental data obtained as part of a larger on-going study investigating changes in cytokines and hormones in GWI and CFS groups was used as a basis for comparison with the predicted resting states. Previous work with the CFS datasets by Broderick et al. presents full repeatability statistics on the cytokine panels using n = 9 CFS, and n = 12 controls [46]. Significant changes in correlation patterns linking immune gene sets in CFS with n = 39 CFS and n = 35 controls) [47]. Similarly in GWI we observed significant changes in association patterns (mutual information) for n = 9 GWI and n = 11 controls across 3 points in time [48] for salivary cortisol and plasma, serum or culture supernatants expression of neuropeptide Y (NPY), IL-1a, IL-5, IL-6, IL-10, TNF-α, IFN-γ and soluble CD26 (sCD26). Larger sample sizes are used in this work to further improve the statistical power and resolution in identifying characteristic differences between subject groups.

#### GWI

Cytokine profiles and endocrine measures were obtained for 27 GWI and 29 HC subjects recruited from the Miami Veterans Administration Medical Center. Subjects were male with an average age of 43 years and BMI of 28. Inclusion criteria was derived from Fukuda et al. [49], and consisted in identifying veterans deployed to the theater of operations between August 8, 1990 and July 31, 1991, with one or more symptoms present after 6 months from at least 2 of the following: fatigue; mood and cognitive complaints; and musculoskeletal complaints. Subjects were in good health prior to 1990, and had no current exclusionary diagnoses [50]. Collins et al. [51] supports the use of the Fukuda definition in GWI. Control subjects consisted of gulf war era sedentary veterans and were matched to GWI subjects by age, body mass index (BMI) and ethnicity. Additional details regarding this cohort and the laboratory assays performed are available in Broderick et al. [48]. Data will be made freely available upon request.

#### CFS

Levels of cortisol (CORT) and estradiol (EST) measured in peripheral blood were obtained from the Wichita Clinical dataset [52] for a group of 39 female CFS subjects and 37 Healthy controls (HCs) with an average age of 52 years and an average body mass index (BMI) of 29. Additional details of this cohort and the laboratory assays performed may be found in work previously reported by our group [53], [54]. Multiplex cytokine profiles were obtained in plasma from a separate but demographically comparable cohort of 40 female CFS subjects and a group of 59 healthy female matched control subjects studied by our group at the University of Miami [55]. Average age in this cohort was 53 years with an average BMI of 26. Profiling of cytokine concentrations was performed in morning blood plasma samples using an enzyme-linked immuno-absorbent assay (ELISA)-based assay. Details of this protocol and results of a comparative analysis of cytokine expression patterns are available in Broderick et al. [55]. In both studies a diagnosis of CFS was made using the International Case Definition [50], [56]. Exclusion criteria for CFS included all of those listed in the current Centers for Disease Control (CDC) CFS case definition, as well as psychiatric exclusions, as clarified in the International CFS Working Group [56]. Data will be made freely available upon request.

### Statistical Analysis

Brown's theoretical approximation [57] of Fisher's statistics was used to calculate the significance of alignment between experimental data and a given model predicted state. Fisher's method, a meta-analysis technique, combines probabilities to obtain the overall significance *P* of a set of p-values obtained from independent tests of the same null hypothesis. The combined *χ^2^* statistic,
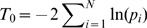
(3)where *N* is the number of measureable variables and *p_i_* is the corresponding p-values under the null hypothesis, has a *χ^2^* distribution with *2N* degrees of freedom assuming that the performed tests are independent. As the molecular variables of the endocrine and immune system interact with one another, as evidenced by the above connectivity diagrams, they are not independent. As a result, direct application of this test statistic is invalid, since the assumption of independence is violated. Brown [57] suggested a method for combining non-independent tests. If the tests are not independent, then the statistic *T_0_* has mean *m* = 2*N* and variance (*σ^2^*) given as,
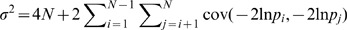
(4)where *p_i_* and *p_j_* are the p-values for each test and the covariance (cov) is calculated as,
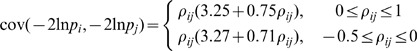
(5)with *ρ_ij_* being the unadulterated correlation between variable *i* and variable *j*. Finally, the overall significance *P* of a set of non-independent tests is calculated using the statistic *T* which under the null hypothesis follows the central χ^2^ distribution, where *T = T_0_/c* with *2N/c* degrees of freedom and *c = σ^2^/4N*.

Here, we test if each experimental measure aligns with a given model predicted state. Our null hypothesis is that the experimental measures do not align. p-values for individual variables, *p_i_*, are calculated using two-sample t-tests between ill subjects and healthy controls. Where model predictions give a variable as high (+1), ‘right-handed’ one-tailed test are used, whereas a ‘left-handed’ test was used when model predictions are low (−1), to give the probability of obtaining the predicted value when the null hypothesis is true. For the case where the model predicts normal behavior for a variable (0) a two-tailed t-test is used. However, the p-value from the two-tailed test, *p_two-tail_*, gives the probability that there is an observable difference between illness and control, which is the null hypothesis. To rectify this, when comparing to a model predicted variable of 0 we take the p-value to be *p_i_* = 1 − *p_two-tail_*, giving the probability of obtaining the predicted value when the null hypothesis is true.

All cohort data was normalized using a Log2 transformation before T-tests and correlation calculations were performed. The unadulterated correlation values *ρ_ij_* between two variables *i* and *j* were calculated in healthy subjects as the pairwise Pearson's linear correlation coefficient between variables. The above-mentioned experimental data was compared against model predictions based on the five measureable variables, namely TEST/EST, CORT, IIR, T1Cyt, and T2Cyt. Where model variables represent an aggregate set of markers each experimentally measured constituent marker was compared individually to the model predicted value. For example, T1Cyt is composed of IL-2, IFNγ and TNFβ, therefore 3 individual p-values were calculated based on the predicted value of T1Cyt.

These significances of alignment are best visualized with a Sammon projection of the aggregated *P* value as distances between the clinical data and the predicted model values. Non-metric multidimensional scaling using Sammon's nonlinear mapping criterion was used to project the *P* value distances onto a 2 dimensional plot. The aggregate *P* values between predicted model states were determined via Brown's method above. Where values between predicted states were found to disagree p-values for individual variables, *p_i_*, were taken as 1. Where values between predicted states were found to agree, *p_i_* was assigned a standard minimum value for significance of 0.05 to avoid numerical instability in the calculation of *T_0_*.

## Results

### Stable States in the HPA Models

**Figure 2:**
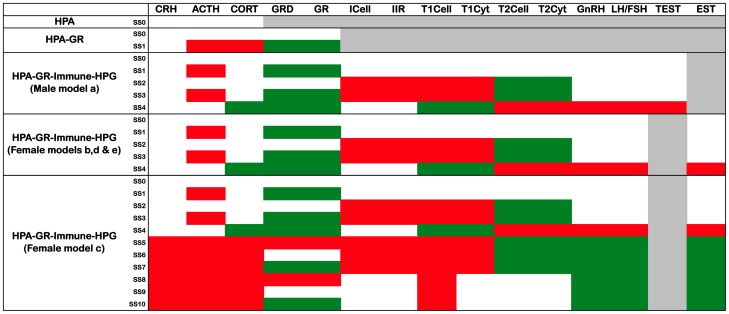
Steady states of standard and extended HPA models. White – nominal state (0); Green – high state (1); Red – low state (−1); Grey – N/A to the model.

Application of the discrete state representation to the basic stand-alone HPA model (Figure 1 A) generated 27 system states, and failed to produce multiple stable resting states (Figure 2). The only stable state found consisted of baseline values (SS0) for all state variables. This result is consistent with previous ordinary differential equation models based on this minimal representation of the HPA axis that produce solutions converging to a single fixed state [18].

Discrete state representation of the HPA-GR model (Figure 1 B) generated 243 system states. Of these, 2 system states possessed no outbound edges and were stable attractor steady states (Figure 2). In the first steady state all state variables assumed nominal values (SS0) whereas the second steady state corresponded to activation of state variables GRD and GR with suppression of ACTH and CORT (SS1). This hypocortisolic solution is consistent with that obtained by analysis of the ordinary differential equation model of the HPA-GR system proposed by Gupta et al. [22] and Ben Zvi et al. [16]. Further models by Walker and colleagues, based on Gupta et al. [22], have shown natural oscillatory rhythms in the HPA axis [19]–[21]. While these models make the significant association between delayed feedback and ultradian rhythms, the oscillations around baseline are small and consistent, representing a single resting behavior. As our discrete state representation considers normal resting values as 0, with −1 and 1 representing significant perturbations, minor deviations are not captured and therefore such small oscillatory behavior can be considered similar to our nominal steady state (SS0). This lower resolution allows for a greater breadth of study, accommodating the inclusion of multiple systems of interest, which previously have not been considered in modeling studies.

Combining the HPA-GR axis with the HPG axis and immune system (Figure 1 B–G) altogether produced 4,782,969 system states. For the male HPG (model a) (Figure 1 C), and three of the four female HPG models (models b, d and e) (Figure 1 D, E, G) five steady states were identified (Figure 2). One stable state is characterized by nominal values for all variables (SS0), which corresponds to the typically normal resting state of the system. The first alternate state (SS1) displays low ACTH with high GRD and GR, while the second (SS2) has inhibited innate and Th1 immune responses (low ICell, IIR, T1Cell, and T1Cyt), with increased Th2 activity (high T2Cell and T2Cyt). The third stable state (SS3) appears to be a combination of SS1 and SS2 with low ACTH, ICell, IIR, T1Cell and T1Cyt, and high GRD, GR, T2Cell and T2Cyt. The final state (SS4) presents with hypercortisolism, suppressed TEST and a shift towards the Th1 immune response (low T2Cell, T2Cyt, GnRH, LH/FSH and TEST/EST, and high CORT, GRD, GR, T1Cyt and T1Cell). The persistently low CORT state seen in the previous stand-alone HPA models of Gupta et al. [22] and Ben Zvi et al. [16] was not recovered here. Instead, CORT was expressed at a nominal or high value for all predicted states. SS1 most closely resembles the results of Gupta et al. [22], and Ben Zvi et al. [16], however these previous models only considered a single regulator of CORT, namely ACTH. The lack of a predicted hypocortisolic state in SS1 here can be attributed to the interplay of multiple regulators of CORT (ACTH, IIR, TEST/EST, and T1Cyt). Inclusion of additional regulators is not expected to further alter this state.

In the final female HPG model (model c) (Figure 1 F), corresponding to the ovulation phase, these same five states were recovered along with six new additional states (Figure 2). In the first three additional states the HPA axis and innate immune response are suppressed with low CRH, ACTH, CORT, ICell and IIR, while the HPG and anti-inflammatory response are raised with high T2Cell, T2Cyt, GnRH, LH/FSH and EST. The difference between the three states is noted in the level of glucocorticoid receptor response, GR and GRD, which together take values of low (SS5), nominal (SS6) and high (SS7). The remaining three additional states all give suppressed HPA (CRH, ACTH, and CORT) and lowered T1Cell activity, with high HPG activity (GnRH, LH/FSH and EST), and are again differentiated by their glucocorticoid receptor levels (GR, GRD): low (SS8), nominal (SS9) and high (SS10).

Overall, inclusion of the simplified immune system and the HPG works to regulate CORT levels in the HPA axis. The male HPG (HPG model a), and the majority of female HPG configurations (HPG models b, d and e), serve to produce either nominal values of CORT, with the potential of a shift towards Th2 activation (SS2 and SS3), or a hypercortisolic state with low TEST/EST and a shift towards Th1 (SS4). Only connections associated with the female gender (HPG model c) were responsible for the emergence of a natural hypocortisolic state (SS5 – SS10). This hypocortisolic state comes with high EST and may have a shift towards Th2 activation in the immune system.

### Comparison of GWI and CFS to Predicted States

Application of Brown's meta-analysis method allowed for the calculation of a combined P-value comparing the experimental data with the predicted stable states, allowing for the alignment between different predicted stable states to be ranked. As experimental measures allowed for comparison with only five variables (TEST/EST, CORT, IIR, T1Cyt, and T2Cyt) several of the predicted stable states resulted in the same experimental profile and resulting combined P-value despite being distinct states (e.g. SS0 and SS1 both show nominal values for the five measureable variables).

**Figure 3:**
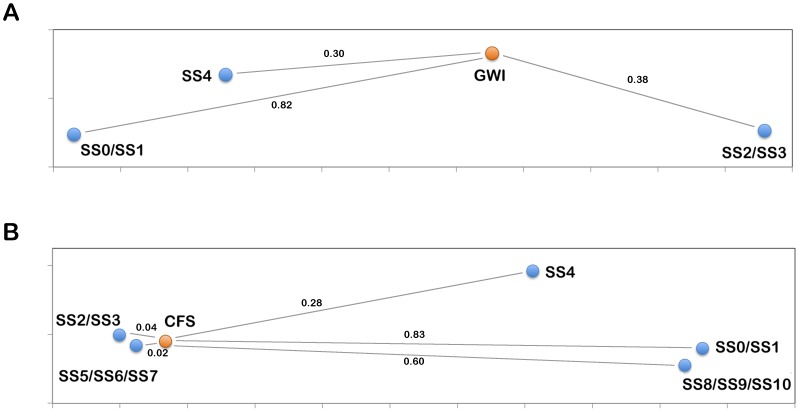
Sammon projection of illness and model predicted states. Axes represent arbitrary units such that the relative Euclidean distance between points approximates the significance of alignment P-value between states, as shown by connecting lines. (A) Male GWI. (B) Female CFS.

To compare to our model the difference between steroid and cytokine levels recorded in male Gulf War veterans with GWI and HCs were compared to the steady state values predicted by the male variant of the HPA-GR-Immune-HPG model (model a). Comparison to the nominal states (SS0/SS1) showed poor alignment, P_SS0/SS1_ = 0.82, suggesting that the GWI profile cannot be considered the same as nominal behavior. Alignment with states presenting a shift towards Th2 immune activation (SS2/SS3) showed better alignment, P_SS2/SS3_ = 0.38, although with low significance. The final state, displaying hypercortisolism, low TEST and a shift towards Th1 immune activation (SS4), yielded the best alignment, P_SS4_ = 0.30, again however, with a low overall significance. These alignments are illustrated via the Sammon plot shown in Figure 3A.

The difference between steroid and cytokine levels of female CFS subjects and HCs were compared to the steady state values predicted by the female variants of the HPA-GR-Immune-HPG models (model b-e). Again, alignment with states presenting nominal changes in measureable variables (SS0/SS1) was poor, P_SS0/SS1_ = 0.83, supporting that CFS is distinctly different from normal behavior. The Th2 shifted immune profile states (SS2/SS3) showed a significant alignment, P_SS2/SS3_ = 0.04, suggesting Th2 activation in CFS. This is further supported by low alignment with the Th1 immune activated state, with hypercortisolism, and low EST (SS4), P_SS4_ = 0.28. Improved alignment is seen in states with a shift towards Th2, coupled with hypocortisolism, and high EST (SS5/SS6/SS7), P_SS5/SS6/SS7_ = 0.02, suggesting that these features contribute to the CFS profile. This is also supported by low alignment with states only presenting hypocortisolism and high EST with no immune activation (SS8/SS9/SS10), P_SS8/SS9/SS10_ = 0.60. These alignments are visualized in Figure 3B.

## Discussion

The existence of multiple stable states is a prime characteristic of systems incorporating feed-forward and feedback mechanisms, and plays a critical part in guiding the complex dynamics observed in biology. These alternate stable regulatory regimes occur due to the feed-forward and feedback mechanisms within the system and may allow escape routes for survival of an insult and provide support in the medium or long-term to what is equivalent to an uneasy cease-fire or adaptive compromise. Examples of such compromises in functional status in exchange for survival include vasovagal response to decreased blood pressure and syncope (“fainting”) [58]. From an evolutionary perspective it would be advantageous for a pathogen to establish an adaptive relationship with the host. As naturally occurring alternate states of homeostasis are inherently stable, exploiting these regimes could be an advantageous way for a pathogen to establish long-term chronic infection, in essence using the body's own homeostatic drive to maintain the status quo. Deviations of persistent illness profiles from normal homeostatic states argue in favor of the continued presence of an initial aggravating factor or of lasting alterations to the regulatory circuitry imparted by the initial insult [59], [60]. The latter would essentially modify the solution landscape resulting in new natural attractors. To explore this hypothesis, we constructed a simple but integrated model incorporating three of the body's major regulatory axes: the HPA, the HPG and the immune system. Modeling the dynamic properties of these complex systems presents a significant challenge, as much of the detailed information describing in vivo kinetics in humans is unavailable. However, there is a very significant body of connectivity data describing the interactions between the molecular and cellular elements of these biological systems. To make use of this wealth of information we have applied a discrete state representation to the neuroendocrine immune system based solely on the biological connectivity found in the literature and a set of ternary logical rules. Using a discrete logic methodology proposed by Thomas [24], we demonstrated that the inclusion of feed-forward/feedback loops leads to multiple stable states. Indeed, addition of the positive feedback loop regulating glucocorticoid receptor dimerization (GR-GRD) to a basic model of the HPA axis generated an alternate homeostatic state characterized by high receptor expression and low circulating cortisol levels, a result found previously by Gupta et al. [22] and Ben Zvi et al. [16] using differential equation based models. So dependent is the natural emergence of these states on the regulatory wiring that inclusion of this receptor dimerization in a more complex HPA-Immune-HPG models resulted in the disappearance of this alternate hypocortisolic state through compensatory effects of these axes. Only when all three interacting axes were included was an alternate hypocortisolic condition recovered. Therefore while simple models require the inclusion of positive receptor feedback dynamics to produce multistability, these effects become inherent in more coarse, but comprehensive regulatory circuits, and receptor-level feedback becomes less of a contributor in the support of multiple attractor states. Coarse-grained but comprehensive models may suffice therefore in capturing physiologically relevant and clinically verifiable response dynamics.

Our analysis of these coarse grained models spanning across multiple regulatory axes highlighted the important role of gender in supporting a persistent hypocortisolic condition. Due to the suppressive actions of the male gonadal system in regulating itself and the HPA axis, a low cortisol steady state is never available to the male, at least theoretically at this level of detail. In women however, the combined effect of EST and PROG on the HPA still remains somewhat inconsistent [28], [61] owing to the varying effects of these hormones during and after the menstrual cycle. EST is generally believed to stimulate the HPA axis during the menstrual cycle [61]-[63], however evidence indicates that in perimenopausal, menopausal or ovariectomized women the HPA axis response is inversely correlated with plasma EST levels suggesting an inhibitory effect [63], [64]. This suggests that sex hormone regulation may change in feedback polarity and act as both inhibitor and activator of the HPA axis. For this reason HPA-HPG interaction in women will in theory readily support the presence of a stable hypocortisolic condition when HPG axis regulation inhibits the HPA axis while stimulating itself.

In addition to sex hormone regulation, interaction with the immune system also appears to play a significant role in determining abnormal cortisol levels. In our coarse-grain models, cortisol exerts a suppressive action on the innate immune system and the Th1 adaptive immune response. Conversely, positive feedback by certain components of the immune system promotes increases in cortisol levels, which support a hypercortisolic steady state. While, inclusion of the glucocorticoid receptor dimerization (GR-GRD) in these models yielded additional steady states, it did not result in any significant changes to the profile in regards to cortisol levels. Combining the actions of HPA, HPG and immune regulation supported the existence of a stable hypercortisolic state in all models of men and women while a persistent hypocortisolic state was available only in women and only under certain modes of HPG regulation. Once again, while the inclusion of the GR-GRD receptor dimerization in this overarching model yielded additional steady states, it did not result in any significant changes to the homeostatic profiles.

These findings suggest that abnormally high levels of cortisol and adaptive immune activation, in this case Th1, may be perpetuated under certain conditions by the system's own homeostatic drive. This prediction of persistent and stable Th1 activation is consistent with evidence of anomalies in immune signaling in GWI [48], [65], [66]. Skowera et al. measured intracellular production of cytokines in peripheral blood and found ongoing Th1-type immune activation in symptomatic Gulf War Veterans compared to healthy counterparts [65]. More recent work confirmed this finding while also suggesting that this may occur in the more complex context of a mixed Th1:Th2 response [48], something not captured by the simple immune model used here. Though we were unable to find documented reports of lower testosterone levels in GWI beyond the experimental data presented here, a large study of gulf war veterans in the UK found increased risk of fertility problems in this population [67], suggesting a possible relation.

In much the same way, conditions involving hypocortisolism and a Th2 shift may also be perpetuated at least in part by the natural homeostatic regulatory programming. In this case the homeostatic program may be driven by sex steroid suppression of the HPA axis and promotion of HPG function coupled with the mutual inhibition between the Th1 response and function of the gonadal axis, a configuration seemingly available only to female subjects in our models. This would suggest that the hypocortisolism seen in diseases, such as CFS [68]–[70], could be a result of the complexity afforded by the interaction between the HPA, immune and HPG axes in female subjects. Indeed model predictions describing such an alternate homeostatic state in women aligned with our experimental results from CFS subjects, and is consistent with previous findings of Th2 activation in CFS [55], [71]–[73]. This alignment with a naturally occurring homeostatic conditions may explain, at least in part, the biased prevalence of such persistent diseases in women [74]–[79]. Indeed, these authors report that approximately 70% of observed CFS patients are women. Additionally, the prevalence of CFS in the 40–49-year-old age range [79], and the higher prevalence of gynecological conditions and gynecological surgeries in women with CFS [80] supports the evidence that HPA suppression by estradiol appears more likely in perimenopausal, menopausal or ovariectomized women [63], [64]. Interestingly, as many as 1 in 3 CFS subjects have reported symptom relief during pregnancy [81]. The normal trend in pregnancy towards increased cortisol levels, especially in the third trimester, might be a contributing factor that would support the key involvement of sex hormone regulation proposed by our analysis [82]. While, in normal pregnancy this increase in cortisol typically coincides with an increase in cortisol-binding globulin (CBG) maintaining the level of free cortisol, CBG genetic variants in CFS have the potential to alter normal CBG function [83], [84].

While certainly more comprehensive than their predecessors, these models remain relatively coarse representations of the interplay between the endocrine and immune systems.

This is particularly true of immune model granularity, especially when one considers the complex signaling network supported by immune cells as well as other immune-sensitive cells [85]. The important role of key neurotransmitters linking the central nervous system with the HPA axis and the immune system was also under-represented in this first generation of models. For example, norepinephrine and epinephrine stimulate the β_2_-adrenoreceptor-cAMP-protein kinase A pathway inhibiting the production of Th1/proinflammatory cytokines and stimulating the production of Th2/anti-inflammatory cytokines causing a selective shift from cellular to humoral immunity [86], [87]. Additionally, lymphocytes express most of the cholinergic components found in the nervous system. Lymphocytes may be stimulated by, or release, acetylcholine thus constituting an immune regulating cholinergic system secondary to the nervous system [88]. Another neurotransmitter, neuropeptide Y (NPY), also serves as a powerful immune modulator [89] and has recently been shown to play a role in CFS [90]. These components are without question important, however based on our initial observations from this piecewise analysis we expect that increased detail will lead to the emergence of additional response programs rather than the elimination of attractors found here.

As these models are based on currently documented knowledge of human physiology and regulatory biochemistry they are necessarily incomplete. Nonetheless the simple models presented here illustrate the importance of an integrative approach to understanding complex illnesses. Further refinement of the model to include more detailed description of interactions within and between the HPA, HPG and immune systems could extend its applicability to other illnesses as would the incorporation of other key systems such as the brain and central nervous systems. Yet, even with the coarse-grained co-regulation networks investigated we found numerous stable resting states that differ significantly from normal and were indicative of complex and persistent regulatory imbalances. Findings such as this support the use of an alternate model for disease, one which is not necessarily associated with failure of individual components, but rather with a shift in their coordinated actions away from normal regulatory behavior. Response to exercise and other stressors has the potential to be very different in these new regulatory regimes. This is something that we have observed firsthand in our work with human GWI and CFS subjects [91].

Finally, when considering alignment with the experimental data presented here for CFS and GWI, it is important to remember that it was never our hypothesis that these illnesses resulted solely from the actions of homeostatic drive. Instead we proposed that homeostatic drive might be a significant contributor to the persistence of illness mechanisms. Because these naturally occurring regimes, once instantiated, provide an alternate stable homeostasis resistant to change, it may offer fertile ground in support of many chronic pathological processes. The alignment of several immune and endocrine markers modeled here with experimental data from CFS and GWI, two chronic conditions, would support at least partial involvement of the body's own homeostatic drive in facilitating the perpetuation of these conditions. Correlation between these illness conditions and predicted stable states does not imply causation. These results do not suggest that homeostatic drive is the root cause of GWI or CFS, only that it might serve to sustain these chronic illnesses. This may promote resistance to therapy and the natural regulatory barrier to change, even positive change, should at least be considered in the design of robust treatment avenues. Knowledge of the basins of attraction identified by the modeling methods presented here can provide a comprehensive overview of multisystem dysregulation. This knowledge may be used to identify multiple therapeutic targets to be treated in conjunction to correct overall system imbalance. This opens the possibility of discrete interventions targeting multiple systems where treatment could eventually be discontinued, leaving normal regulatory drive to return the system to the correct resting state. This is very different from conventional long-term administration of a drug regimen, whereby the system is held artificially in a more desirable, but unstable state through continued intervention.
