# Correction: A Role for Homeostatic Drive in the Perpetuation of Complex Chronic Illness: Gulf War Illness and Chronic Fatigue Syndrome

**DOI:** 10.1371/journal.pone.0094161

**Published:** 2014-04-03

**Authors:** The PLOS ONE Staff

An error occurred in Equation 2 causing “…” to be replaced with a double arrow. Please see the corrected Equation 2 here:
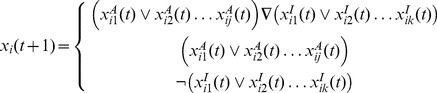
(2)


## References

[1] Craddock TJA, Fritsch P, Rice MA Jr, del Rosario RM, Miller DB, et al (2014) A Role for Homeostatic Drive in the Perpetuation of Complex Chronic Illness: Gulf War Illness and Chronic Fatigue Syndrome. PLoS ONE 9(1): e84839 doi:10.1371/journal.pone.0084839 24416298 10.1371/journal.pone.0084839 PMC3885655

