# Correction: A Role for Homeostatic Drive in the Perpetuation of Complex Chronic Illness: Gulf War Illness and Chronic Fatigue Syndrome

**DOI:** 10.1371/journal.pone.0100355

**Published:** 2014-06-16

**Authors:** The PLOS ONE Staff

There are a number of errors in Figure 1. Please see the corrected Figure 1 here.

**Figure 1:**
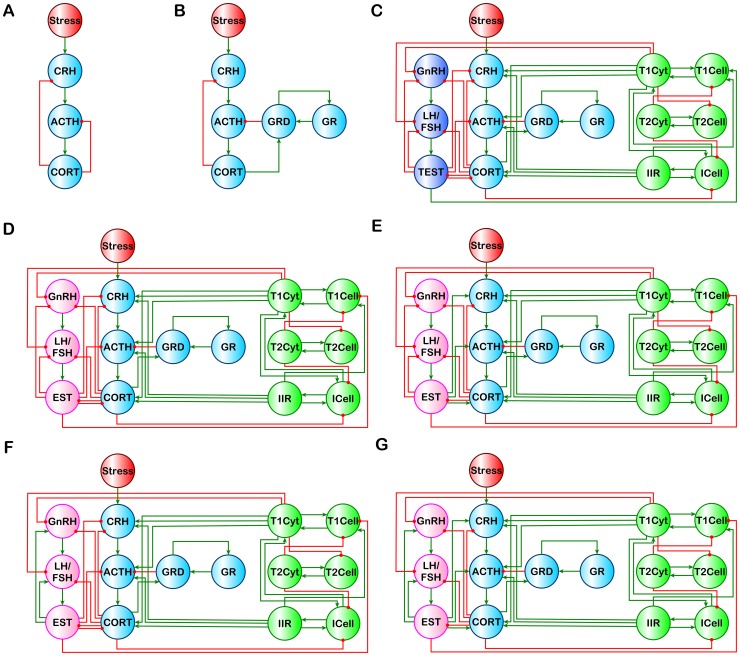
Standard and extended HPA models. (A) Standard HPA model. (B) HPA-GR model of Gupta et al. [22]. Integrated models (C) HPA-GR-Immune-HPGa for males, and (D) HPA-GR-Immune-HPGb, (E) HPA-GR-Immune-HPGc, (F) HPA-GR-Immune-HPGd, and (G) HPA-GR-Immune-HPGe for females. For (C) – (G) connections between the sex steroid EST and the HPG and HPA components change between stimulatory and inhibitory to capture the effects of the menstrual cycle.
